# The COVID-19 Infodemic: A Quantitative Analysis Through Facebook

**DOI:** 10.7759/cureus.11346

**Published:** 2020-11-05

**Authors:** Naseem Ahmed, Tooba Shahbaz, Asma Shamim, Kiran Shafiq Khan, S.M. Hussain, Asad Usman

**Affiliations:** 1 Pathology, Dow University of Health Sciences (DUHS), Karachi, PAK; 2 Medicine, Dow University of Health Sciences (DUHS), Karachi, PAK; 3 Internal Medicine, Dow University of Health Sciences (DUHS), Karachi, PAK

**Keywords:** misinformation, facebook, covid-19, pandemic, coronavirus

## Abstract

Background

Social media is a crucial part of our daily life. Facebook, being the biggest social media platform, plays a significant role in the spread of information influencing the global response to the COVID-19 pandemic. Health care agencies like the World Health Organization (WHO) and Centers for Disease Control and Prevention (CDC) use social media as a platform to impart information regarding COVID-19; simultaneously, there is a spread of misinformation on social media, masking the credible sources of information. Our research aims to assess the utility of Facebook in providing misinformation and testing its “fact-check policy.”

Methods

An online search was conducted on Facebook by a newly created account to eliminate bias. The Facebook search bar was used to investigate multiple keywords. Data were tabulated in Microsoft Excel (Microsoft Corporation, Redmond, WA). Descriptive statistical analysis of Facebook accounts and posts was done using the Statistical Package for the Social Sciences (SPSS) version 26 (IBM Corp., Armonk, NY) while statistical importance was set a priority at a p-value of 0.05.

Results

Our study consisted of 454 Facebook posts. Most (22.5%) were posted by verified accounts and 23.9% by informal individual/group accounts. The tone for most (40.4%) COVID-19 information was serious while the most common (43.9%) topic was medical/public health. In total, 22.3% included misinformation, 19.6% were unverifiable, and 27.5% included correct information verifiable by the WHO or CDC.

Conclusions

Misinformation/unverifiable information related to the COVID-19 crisis is spreading at a distressing rate on social media. We quantified the misinformation and tested Facebook’s “fact-check policy.” We advise strict initiatives to control this infodemic and advise future researches to evaluate the accuracy of content being circulated on other social media platforms.

## Introduction

Social media has become a crucial component of our everyday life in today’s globalizing society. It has a penetration of 40.9% of the entire world population, and it is estimated that, globally, 2.95 billion individuals are using social media as of 2019 [1]. Facebook has become the biggest social media platform universally since around 2.6 billion monthly active users were reported in the first few months of 2020, [2]. Therefore, social media, specifically Facebook, plays a significant and crucial role in the spread of information across the world, influencing the global response to this pandemic.

Coronavirus disease 2019 (COVID-19) causes a severe respiratory illness similar to severe acute respiratory syndrome [3]. Recent evidence shows that the COVID-19 virus spread to human beings through transmission from wild animals that were illegally sold in the Huanan seafood market [3]. Phylogenetic analysis shows that the COVID-19 virus is a new member of the Coronaviridae family but is separate from severe acute respiratory syndrome coronavirus (SARS-CoV) and Middle East respiratory syndrome coronavirus (MERS-CoV) [3]. The typical symptoms of COVID-19 are cough, dyspnea, sore throat, fatigue, and, most commonly, fever that occurs soon after exposure to a carrier. It may lead to pneumonia and severe disease as well, especially for the elderly [3].

In this modern era, where technology is a click away, healthcare agencies like the World Health Organization (WHO) [4] and the US Centre of Disease Control and Prevention (CDC) [5] use social media as a platform to impart up-to-date information regarding COVID-19. Simultaneously, numerous rumors, misinformation, myths, and hoaxes have appeared on social media too, consequently drowning our credible sources of information, which have collectively received only some hundred thousand engagements [6]. Whether well-intentioned or malevolent in nature, this plethora of misinformation leads to a fear of an otherwise low-mortality infection; inappropriate prescribing and overdosage of harmful drugs, decreasing healthy behaviors, and promoting unfitting practices, hence resulting in suboptimal control of the COVID-19 crisis across the globe [7].

To scrutinize the reliability of its content, Facebook uses the International Fact-Checking Network’s Code of Principles to choose fact-checking partners from all over the world [8]. When any data is considered incorrect, Facebook alerts users who have recently interacted with the said post and then reduces that post’s visibility to other Facebook users [9]. Despite these efforts, it was flooded with misinformation, forcing Facebook to announce updates and stricter policies in March 2020 [8].

Keeping this infodemic challenge in mind, our research aims to assess the utility of Facebook in providing misinformation, unverified information, and correct information regarding the coronavirus, concurrently testing the updated fact-check policy of Facebook.

## Materials and methods

**Figure 1:**
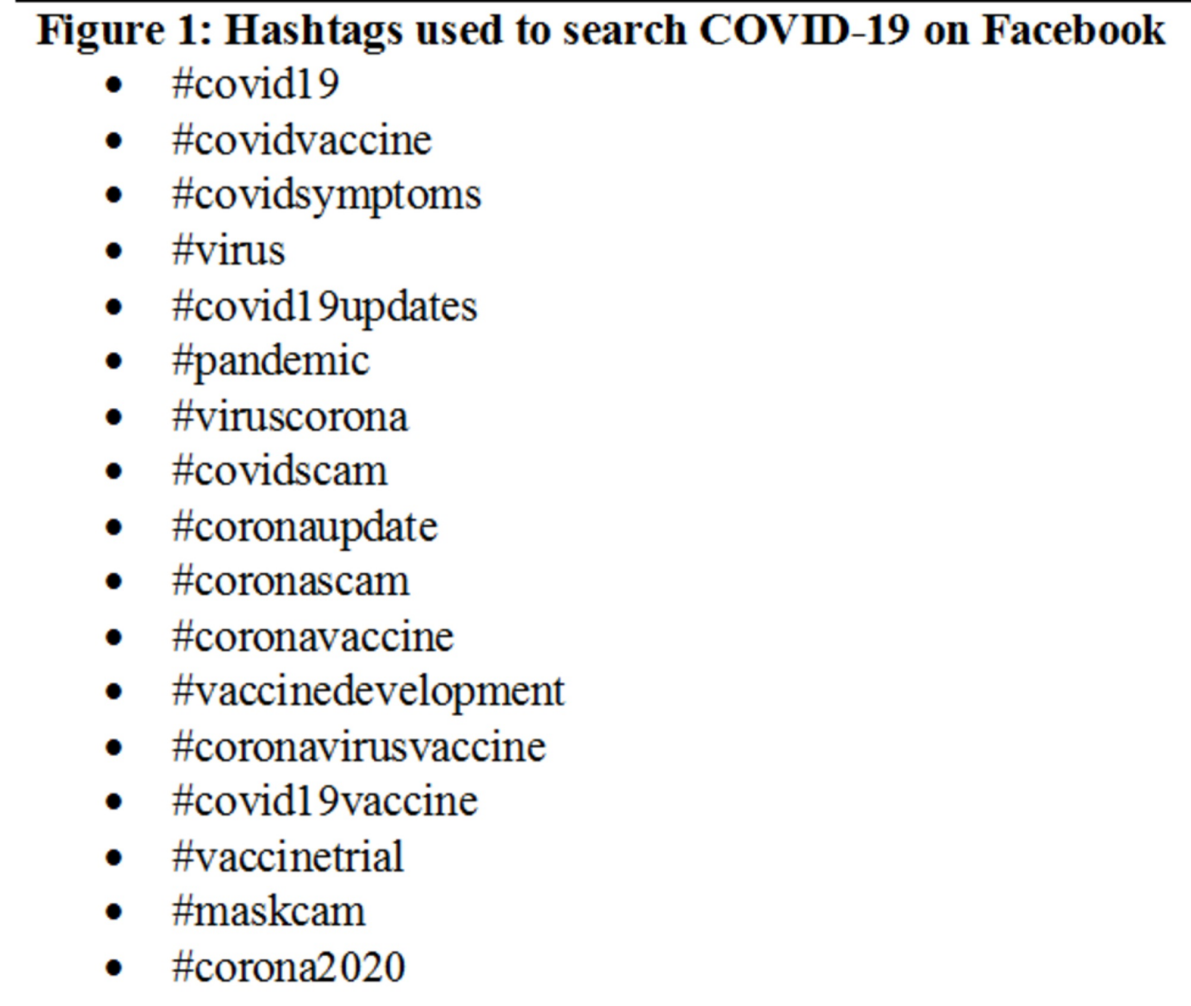
Hashtags used to search COVID-19 on Facebook

We conducted web-based research using the Facebook platform from July 14, 2020, to July 16, 2020. A newly created account was used to eliminate bias in the search results. We used the Facebook search bar using multiple keywords, as mentioned in Figure 1. Our search was restricted to posts in the English language only and to those having a minimum of 10 likes. We eliminated posts that had less than 10 likes Data of all the posts in the results of the searched hashtag were collected that included any information relating to coronavirus and were tabulated in Microsoft Excel (Microsoft Corporation, Redmond, WA). Our research did not require the approval of the institutional review board (IRB) since all the data that we collected was from public accounts.

Each post was categorized as correct information, misinformation, or unverifiable information. They were cross-checked with the information provided by the updated guidelines of the World Health Organization (WHO) and the Center for Disease Control and Prevention (CDC) [4-5]. Those corresponding to the information were characterized as correct information. Posts that included any content that could be easily be rebutted using any of the aforementioned sites were considered misinformation while we categorized unverifiable information as posts that could not be proven either correct or incorrect by the same sources.

For each individual post, a couple of predetermined variables were collected by the authors regarding their content and account characteristics. Accounts were categorized into the following categories: nongovernmental organization (NGO), government, news outlet, healthcare, and informal individual/group. Account verification was also charted. A verified account is defined as one of public interest that is deemed to be authentic by Facebook. The contents of each post were classified according to its tone and topic. The categorization of topics was health, financial, and sociopolitical. The tone of content was categorized into the following: serious, humorous, and opinion. Posts categorized as serious were those containing information related to COVID-19, posts labeled as opinion were those that portrayed the account's own point of view and humorous posts were those containing memes or jokes.

Statistical analysis

Descriptive statics were taken to analyze Facebook accounts and post characteristics. Chi-square statistics were utilized to determine p-values for the relation between accounts/posts characteristics and the presence of misinformation, unverifiable information, and correct information. Bar graphs were created using Microsoft Office Word. Statistical importance was set priority at a p-value of 0.05. All analysis was executed using the Statistical Package for the Social Sciences (SPSS) version 26 (IBM Corp., Armonk, NY).

## Results

Accounts and post characteristics

Our study consists of a total of 454 Facebook posts. Most of them were posted by verified accounts (291, 44.5%), followed by informal individual/group (156, 23.9%). Of all the accounts, the least was posted from NGOs (30, 4.6%) and government accounts (12, 1.8%) (Table 1). In Table 2, we present the characteristics of the individual posts. The bulk of information regarding COVID-19 included serious content (264, 40.4%) while only 84 (12.8%) posts contain humorous content. The most common topic was medical/public health (287, 43.9%), followed by socio-political (96, 14.7%) and financial (68, 10.4%).

**Table 1:** Facebook account characteristics NGOs: nongovernmental organizations

Characteristics	N (%)
Verified Facebook account	291(44.5%)
Informal individual/groups	156(23.9%)
Healthcare/public health	130(19.9%)
News outlet/journalist	114(17.4%)
NGOs	30(4.6%)
Government	12(1.8%)

**Table 2:** Individual post characteristics

Characteristic	N (%)
Tone	
Serious	264 (40.4%)
Humorous/non-serious	84 (12.8%)
Opinion	99 (15.1%)
Topic	
Public Health/Medical	287 (43.9%)
Financial	68 (10.4%)
Socio-political	96 (14.7%)

Misinformation, correct, and unverifiable information

**Figure 2:**
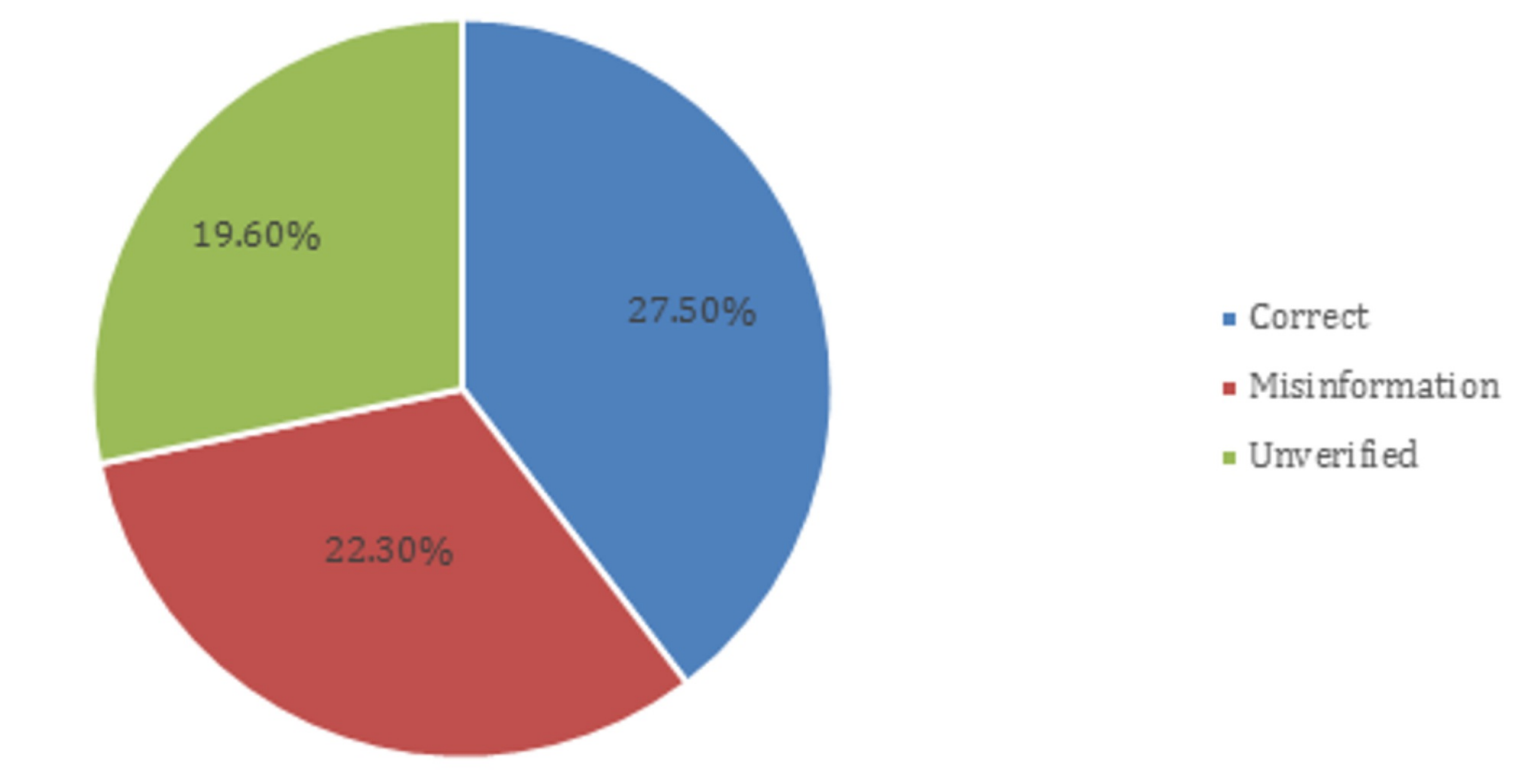
Accuracy of information

In total, 146 posts (22.3%) included misinformation, 128 (19.6%) included unverifiable information and 180 (27.5%) include correct information verified by WHO or CDC (Figure 2). Informal individual/group accounts had more misinformation when analyzed Facebook post by post category (65, 40.1%, p: <0.001) (Table 3). In addition, the same category posted the most unverified information (44, 27.2%). Government, NGOs, news outlets/journalists, and healthcare/public health accounts all had a low rate of misinformation respectively, shown in Table 3. Moreover, Facebook posts posted by verified Facebook accounts included more unverified information when compared to those posted by unverified accounts (unverified account: 26.4%, verified account: 29.2%, p: <0.001) vice versa for posts with false information where misinformation is posted more from unverified accounts than verified (unverified: 40.5%, verified: 27.5%). News outlet/journalist accounts (16.5%) contain the lowest rate of misinformation as compared to other accounts. Furthermore, public health accounts maintain the second-highest record of posting misinformation (51, 38.3%). The number of likes per post and the number of shares per post show no association, with any significant difference in terms of unverifiable and misinformation rates (p>0.05) (Table 3).

**Table 3:** Comparing Facebook post and account characteristics with authenticity of information

Post/Account characteristic	Misinformation, N (%)	p-value	Correct information, N (%)	p-value	Unverifiable information, N (%)	p-value
Healthcare/public health		<0.001		0.076		0.45
Yes	51(38.3%)		53(39.8%)		29(21.8%)	
No	82(61.7%)		80(60.2%)		114(78.2%)	
NGO		0.034		0.001		0.025
Yes	8(28.6%)		11(39.3%)		9(32.1%)	
No	20(71.4%)		17(60.7%)		19(67.9%)	
News outlet/journalist		0.040		0.004		<0.001
Yes	19(16.5%)		52(45.2%)		44(38.3%)	
No	96(83.5%)		63(54.8%)		69(60%)	
Government		0.001		0.07		0.43
Yes	3(80.8%)		10(62.5%)		3(18.8%)	
No	13(81.2%)		6(37.5%)		13(81.2%)	
Informal individual/groups		<0.001		0.98		0.77
Yes	65(40.1%)		53(32.7%)		44(27.2%)	
No	97(59.9%)		109(67.3%)		115(71%)	
Verified account		<0.001		0.07		0.001
Yes	80 (27.5%)		126(43.3%)		85 (29.2%)	
No	66 (40.5%)		54(33.1%)		43 (26.4%)	
No. of likes						
<1000	140(35.6%)	0.067	141(35.9%)	0.01	112(28.5%)	0.065
>1000	6(9.8%)		38(62.3%)		17(27.9%)	
No. of shares						
<500	135(34.5%)	0.077	143(36.6%)	<0.001	113(28.9%)	0.090
>500	11(17.5%)		36(57.1%)		16(25.4%)	

**Figure 3:**
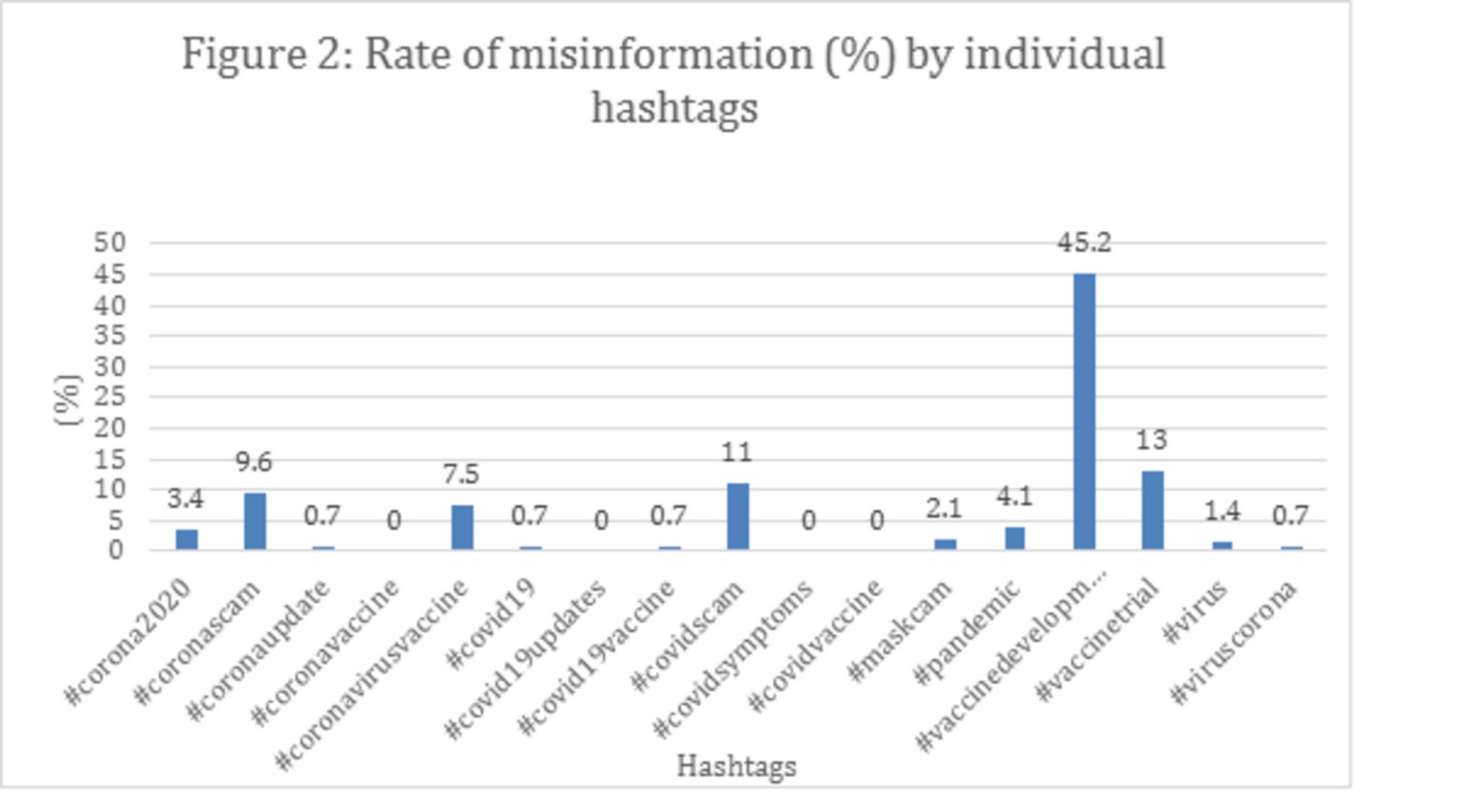
Rate of misinformation (%) by individual hashtags

**Figure 4:**
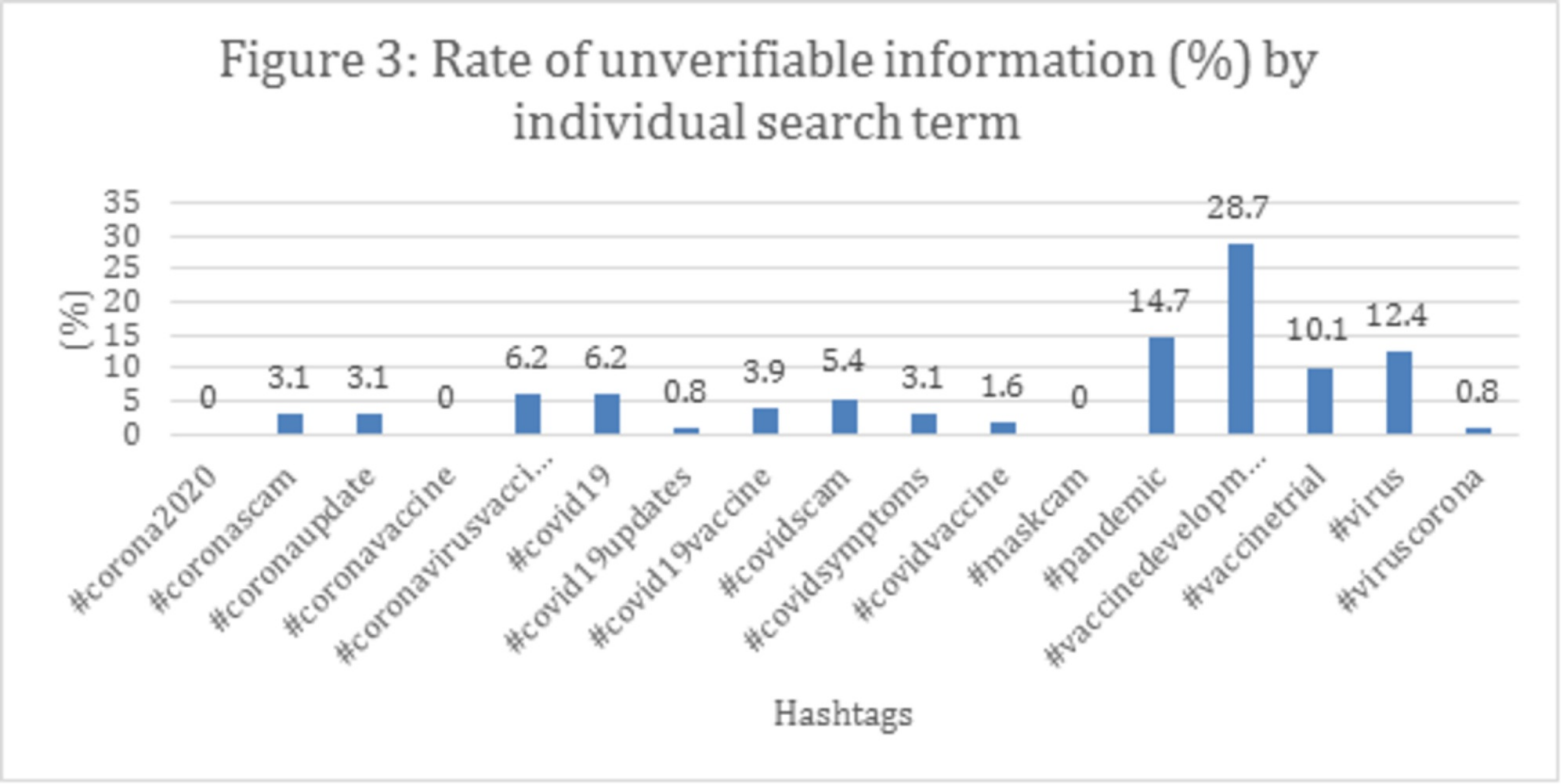
Rate of unverifiable information (%) by individual search term

Accounts with a higher number of shares and likes contain correct information (62.3%, 57.1%, respectively) demonstrating that more likes and shares are associated with correct information (p<0.01). Conclusively, the frequency of misinformation varied among hashtags, presenting that the hashtag "#vaccinedevelopment" had the highest rate of misinformation (Figure 3), the hashtag "#vaccinedevelopment" had the highest rate of unverifiable information, while "mask cam" and "#coronavaccine" had the lowest (Figure 4).

## Discussion

The SARs-CoV2 virus emerged from the Hubei province in China in late 2019, but soon, the entire earth got inundated by this viral disease [10], resulting in numerous hospitalizations in the early months of 2020. Focusing on the devastating effects of the virus, WHO declared it a “global health emergency” [11-12]. Soon enough, social media got plagued with colossal amounts of information linked to the virus [13], as the public not only input content on social media but also used it as a means to seek health information and news from over the world. Posts ranged from individual preventive measures (e.g. the effectiveness of masks in containing the contagion [14] to treatment availability (e.g. reports on hydroxychloroquine [15]). This process enabled an explosion of unchecked information and the spread of misinformation. To our knowledge, our research is the first study that uses Facebook as a platform for analyzing the misinformation ratio.

In our study, the total 454 posts on Facebook were posted by accounts owned by healthcare organizations 130 (19.9%), news outlets 114 (17.4%), NGOs 30 (4.6%), governments 12 (1.8%) as well as other informal individuals/groups accounts 156 (23.9%). They all played a massive part in posting information concerning different aspects of COVID-19 with informal individuals/groups (156; 23.9%) posting the highest content. Similar research pertaining to the coronavirus infodemic on Twitter tabulated their results as healthcare/public health 73 (10.8%), news outlets 111 (16.5%), business/NGO/government 37 (5.5%), medical/public health 468 (69.5%), and informal individual/group 448 (66.6%) posting the second-highest content. As serious as this infection is, most of the posts were serious in nature to 264 (40.4%). Coronavirus has not only affected our health but also the socio-political aspects of human lives; similarly, most of the topics of the posts were related to health 287 (43.9%) while some pertained to socio-political topics 96 (14.7%). Nonetheless, posts related to finance (68; 10.4%) also took part in disseminating information, as this virus has a negative impact on job security, resulting in a global economic recession and increased expenditure in terms of safety gadgets like masks, gloves, sanitizers, personal protective (PPE), etc. Conclusively, almost all types of content, whether serious 264 (40.4%), humorous 84 (12.8%), expressing opinion 99 (15.1%) - heath, sociopolitical, or financial-related topics - emerged on Facebook and are constantly being uploaded by accounts of different backgrounds. Similar COVID-19 infodemic data collected on Twitter [16] tabulated different topics and tones of Tweets as follows: serious Tweets (614; 91.2%), humorous or non-serious Tweets (41; 6.1%), opinion (144; 23.0%), public health Tweets (468; 69.5%), Tweets regarding finance (38; 5.6%), and sociopolitical tweets (242; 40.0%). This study also concluded medical health to be the most common topic discussed on Twitter.

Similar to our study, people use Facebook to seek help and educate themselves, however, the “tsunami of information” holds an alarmingly high risk of misinformation and unverifiable information. Misinformation is defined as a “claim of fact that is currently false due to lack of scientific evidence" [17]. Distribution of fallacious content across different social media has become a common practice and there is no exception for the COVID-19 crisis [18]. Finally, on February 15, 2020, the general director of WHO stated that “We’re not just fighting an epidemic; we’re fighting an infodemic” [19]. Social media was the culprit of the dissemination of false news most notably during the 2016 U.S. presidential campaign as well [20]. Another prominent example is the rise of numerous anti-vaccine campaigns when social media became the medium of propagation of inaccurate and harmful information [21]. This has happened over the years since social media and Facebook give the freedom to post any content whether it is verified by scientific evidence or not and the easy access to sharing Facebook posts disseminates such content like wildfire. Our results depict that misinformation and unverifiable information collectively is more than correct information on Facebook, hence verifying the former. Our results are also in line with Gunther Eysenbach’s information wedding cake model where Gunther et al. depict social media as the largest segment of the cake, representing the vast amount of nearly unfiltered and uncontrolled information contributed or amplified by the public [22].

Similar circumstances occurred during the time of the Zika virus epidemic, misguidance, and false news spread. Neeraja et al. in their study stated that on Facebook, accurate information about the Zika virus and its disease was less popular than erroneous videos and posts [23].

The fight against the infodemic is a real challenge, as it spreads very rapidly on social media. Tustin et al. and Xu et al. also reported widespread misinformation about side effects, as well as mistrust in government or pharmaceutical companies in discussions on vaccination [24]. False information over vaccines seems like an incessant trend. A COVID-19 vaccine is still under development, however, as our results interpret, fallacious content relating to it already exists on social media since the hashtag “vaccine development” was associated with a higher rate of misinformation (45.2%) and unverifiable information (28.7%). This suggests a continuous distrust of the public with vaccines, therefore, any future post regarding vaccines should be strictly under observation, whether pertaining to COVID-19 or not. Considering the aforementioned history, we predict that once the anti-COVID-19 vaccine is invented, it might have immense mistrust among the public, with hoaxes and myths spreading across social media, resulting in a large population of people not accepting the vaccine. If strong and clear-cut statements are not made exposing and condemning misinformation, it may have a devastating effect on the public [6]. The COVID-19 crisis already causes increased anxiety and has an unprecedented impact on mental health [25]. False information just adds more fright and unhealthier behaviors. For example, in March 2020, hundreds of Iranian citizens died after ingesting alcohol in a bid to treat COVID-19 as a result of misinformation circulating on social media [26].

Research conducted on the spread of misinformation about Ebola during its epidemic concluded that the quantity of incorrect information was low; similarly, our data collected in the month of July portray that the presence of false and unverifiable information is less than correct information, yet they collectively exist in a significant amount versus correct information. These results are also parallel to those collected on Twitter during the month of February 2020 where misinformation and unverifiable information had a strong relationship with user account verification [27], as our sources of misinformation were also mostly from unverified accounts. Henceforth, Facebook should keep a stricter check on public unverified accounts, as they are a major source of distribution of unverifiable and misinformation.

VP Integrity of Facebook, Guy Rosen, in his updated letter on April 16, 2020, claims that Facebook removes or limits the spread of false information related to COVID-19 that could cause impending harm to the public. They either tag the content as false or remove it completely [28]. They have previously removed false information about rumors of the polio vaccine from Pakistan where it risked the ill-treatment of medical professionals [8]. Fortunately, in our study, the number of likes with correct information (38; 62.3%) and shares with correct information (36; 57.1%) were significantly more than likes with incorrect (6; 9.8%) or unverifiable information (17; 27.9%) and shares with incorrect (11; 17.5%) and unverifiable information (16; 25.4%), implying that the popularity of correct posts was more among users, as they received more engagement from the users. This could be the result of Facebook users not sharing content that was flagged as false or could entirely be because of the reduced visibility of erroneous content to the public. This is in contrast to Xinning et al., according to whom, people retweet jokes and sports events more regarding the Zika virus epidemic especially when those jokes and events include unverified information [23]. Another study’s findings pertaining to the information of vaccines on YouTube by Gabrielle et al. state that videos with positive and correct information were viewed and shared less and had fewer likes than those with a negative tone and material [19]. This may suggest a better content verifying system of Facebook as compared to not only Twitter but also YouTube, which are a couple of the most used social media platforms. Hence, it is safe to say that Facebook’s fact-check policy is succeeding in limiting the spread of false information as compares to other social media.

In a population with a low literacy rate, health-related myths but the mostly increased availability of free time to the general public all over the world as a result of the COVID-19 lockdown may have contributed to the infodemic even more. Health literacy is defined as the individuals’ capacity to obtain, process, and understand basic health information and services needed to make appropriate health decisions and to address or solve a health-related problem. Reports show that a rumor has a three times greater chance to be shared on social media than a verified story. Lack of health literary and health myths, as well as free time, will only amplify the spread of such posts. There’s no ambiguity that it is tough and takes decades to tackle the problem of illiteracy in populations. Also, there is complete uncertainty of the uplifting of lockdowns in various parts of the world. However, it is easier to keep a check on the quality and quantity of information flow on social media, which will, no doubt, ease the panic and control harmful and unhealthy practices.

Despite our promising results showing trends in the inflow of information among Facebook users, our study has a few limitations. First, our study was limited to the English language, which may have generalized our results for non-English speakers. Second, the use of specific hashtags as search terms might have resulted in authors missing those posts that did contain COVID-19 information but didn’t utilize the hashtags while posting their content. However, we selected the most common hashtags, which were a total of 17 in number, greater than any keywords used in previous similar researches carried out on Facebook, and included all the posts resulting from those hashtags for three days [29]. Lastly, our search timeframe was restricted to a few days and, therefore, might not have captured the changing topics that might have advanced with the pandemic. This invites additional research to fill the vital information gap. Nevertheless, our research not only provides timely data but also proves the validity of Facebook’s promising fact-check policy.

As recommended by Gunther Eysenbach’s fourth pillar of infodemic management [22], we also suggest that continuous monitoring and analysis of data and information flow patterns on social media should be undertaken so that outbreaks of misinformation, rumors, and falsehoods could be detected immediately and countered with facts or other interventions like flagging or removing the content from the social media platform, consequently decreasing the dissemination of negative information and panic among the public. Leticia et al. stated that limiting the spread of incorrect information by algorithmic and social corrections is also effective [20]. Content should not only be checked after it has been posted, but it should be verified before it is made visible to the public so that we could cut the cycle of the spread of misinformation before it starts. This should at least be applied over trending topics and posts getting larger engagements, likes, and shares. The quality of information, i.e. correct or incorrect, as well as the quantity and distribution in social media both, should be kept under check. Moreover, social media requires generating metrics not only of information supply but also of information demands, i.e. search queries and hashtags, for better control of any future infodemic.

## Conclusions

Misinformation and unverifiable information pertaining to the worldwide COVID-19 crisis is proliferating at a disturbing rate on social media. We quantified the misinformation spread and tested Facebook’s “fact-check policy.” Our research also provides an initiative for future researches to evaluate the accuracy of content being circulated on various other social media platforms like Instagram, WhatsApp, YouTube, etc., which are widely used by the general public, and study the impact of the propagation of rumors on behavior and precautionary habits that people need to adopt in times of public crises. Interventions from relevant authorities are crucial in order to harness the positive power of social media to distribute accurate and error-free information, as it affects herd behavior. Facebook’s fact-check policy is doing wonders; yet, there still exists a margin for improvement.
